# Malaria Burden Stratification in Malawi- A report of a consultative workshop to inform the 2023-2030 Malawi Malaria Strategic Plan

**DOI:** 10.12688/wellcomeopenres.19110.1

**Published:** 2023-04-19

**Authors:** Donnie Mategula, Collins Mitambo, William Sheahan, Nyanyiwe Masingi Mbeye, Austin Gumbo, Collins Kwizombe, Jacob Kawonga, Benard Banda, Gracious Hamuza, Alinafe Kalanga, Dina Kamowa, Jacob Kafulafula, Akuzike Banda, Halima Twaibi, Esloyn Musa, Atupele Kapito-Tembo, Tapiwa Ntwere, James Chirombo, Patrick, Ken Kalonde, Maclear Masambuka, Lumbani Munthali, Melody Sakala, Abdoulaye Bangoura, Judy Gichuki, Michael Give Chipeta, Beatriz Galatas Adrade, Michael Kayange, Dianne J Terlouw

**Affiliations:** 1 School of Global and Public Health, Kamuzu University of Health Sciences, Blantyre, Malawi; 2 Malawi-Liverpool Wellcome Programme,, Blantyre, Malawi; 3 Liverpool School of Tropical Medicine, Liverpool, L3 5QA, UK; 4 Research Unit, Ministry of Health, Lilongwe, Malawi; 5 PATH, Seatle, Washington, USA; 6 National Malaria Control Programme, Ministry of Health, Lilongwe, Malawi; 7 U.S. President's Malaria Initiative, United States Agency for International Development (USAID), Lilongwe, Malawi; 8 Country Health Information Systems and Data Use (CHISU) Program, Lilongwe, Malawi; 9 Mulanje District Council, Mulanje, Malawi; 10 Nkhotakota District Council, Nkhotakota, Malawi; 11 Department of Mathematical Sciences, School of Natural and Applied Sciences,, University of Malawi, Zomba, Malawi; 12 Kasungu District Council, Kasungu, Malawi; 13 PMI VectorLink Project, Abt Associates, Lilongwe, Malawi; 14 Strathmore University, Institute of Healthcare Management, Nairobi, Malawi; 15 African Institute for Development Policy (AFIDEP), Lilongwe, Malawi; 16 World Health Organisation, Geneva, Switzerland

**Keywords:** Malaria, Malawi, Stratification, Burden, Strategic Plan

## Abstract

**Background: **Malawi's National Malaria Control Programme (NMCP) is developing a new strategic plan for 2023-2030 to combat malaria and recognizes that a blanket approach to malaria interventions is no longer feasible. To inform this new strategy, the NMCP set up a task force comprising 18 members from various sectors, which convened a meeting to stratify the malaria burden in Malawi and recommend interventions for each stratum.

**Methods: **The burden stratification workshop took place from November 29 to December 2, 2022, in Blantyre, Malawi, and collated essential data on malaria burden indicators, such as incidence, prevalence, and mortality. Workshop participants reviewed the malaria burden and intervention coverage data to describe the current status and identified the districts as a appropriate administrative level for stratification and action.

Two scenarios were developed for the stratification, based on composites of three variables. Scenario 1 included incidence, prevalence, and under-five all-cause mortality, while Scenario 2 included total malaria cases, prevalence, and under-five all-cause mortality counts. The task force developed four burden strata (highest, high, moderate, and low) for each scenario, resulting in a final list of districts assigned to each stratum.

**Results: **The task force concluded with 10 districts in the highest-burden stratum (Nkhotakota, Salima, Mchinji, Dowa, Ntchisi, Mwanza, Likoma, Lilongwe, Kasungu and Mangochi) 11 districts in the high burden stratum (Chitipa, Rumphi, Nkhata Bay, Dedza, Ntcheu, Neno, Thyolo, Nsanje, Zomba, Mzimba and Mulanje) and seven districts in the moderate burden stratum (Karonga, Chikwawa, Balaka, Machinga, Phalombe, Blantyre, and Chiradzulu). There were no districts in the low-burden stratum.

**Conclusion: **The next steps for the NMCP are to review context-specific issues driving malaria transmission and recommend interventions for each stratum. Overall, this burden stratification workshop provides a critical foundation for developing a successful malaria strategic plan for Malawi.

## Introduction

Malawi aims to eliminate malaria ^1^. Since the year 2000, there have been four strategic plans that have been implemented. The most recent one started in 2017 and is ending in 2022 and aims to reduce the incidence of malaria from 386 per 1000 population in 2015 to 193 per 1000 by 2022 and to reduce malaria deaths from 23 per 100,000 population in 2015 to 12 per 100,000 by 2022 ^1^.

Over the last two decades, malaria control efforts in Malawi have been scaled up substantially through multiple control measures that include the use of insecticide treated bed nets, Artemisinin Combination Therapies (ACTs), Intermittent Preventive Treatment in pregnancy (IPTP), and malaria rapid diagnostic Tests (mRDTs) ^2, 3^, indoor residual spraying (IRS) and more recently the RTS,S malaria vaccine ^4^. Based on Malaria Indicator Survey (MIS) reports, substantial reductions in malaria transmission have been reported. For example, the national malaria prevalence in 2010 was 44% and subsequently reduced to 10.5% in 2021, representing a 52.2% reduction over 11 years ^5– 7^. Similarly, malaria incidence and mortality have reduced from 407 per 1,000 to 361 per 1,000 population in 2016 and 65.2% decrease in mortality from 23 per 100,000 to 8 per 100000 population in 2021 respectively ^8^.

Variations in intervention coverage and user uptake, weather patterns, vector, host, and parasite factors are changing the malaria landscape leading to pockets of high transmission and low transmission within the same geographical units such as districts, a status referred to as ‘heterogeneous transmission’. In a heterogeneous transmission setting, application of malaria interventions is without geographic targeting at subnational level is less cost-effective. It does not have the impact of reducing malaria impact indicators such as incidence, prevalence, and mortality ^9^. Moreover, in resource limited settings such as Malawi, the scarce resources need to be appropriately allocated considering the observable flattening and reduction in donor as well as government support for malaria control and elimination. The Malawi National Malaria Control Program (NMCP) is currently performing an end-of-strategy review in preparation for developing the 2023 to 2030 malaria strategic plan. In this process, the program is poised to move to a targeted approach in implementing malaria control interventions. Some of the critical reasons for this shift are that there is an observable flattening and reducing donor support for malaria control and elimination. Additionally, the government's allocation of resources for malaria control is also decreasing over time. Recent experiences from the rollout of the RTS,S malaria vaccine serve as an example of the need to shift to a targeted approach. The programme is determined that through better analysis and strategic use of quality data, the country can pinpoint where to deploy the most effective malaria control tools for maximum impact. In 2016, the World Health Organisation (WHO) took a similar approach to malaria control as; approximately 70% of the world's malaria burden was concentrated in just 11 countries – 10 in sub-Saharan Africa (Burkina Faso, Cameroon, the Democratic Republic of the Congo, Ghana, Mali, Mozambique, Niger, Nigeria, Uganda and the United Republic of Tanzania) and India. The sorting of the global cases led to the development of the high burden to high impact (HBHI) approach. The HBHI strategy was introduced to fight malaria in these 11 countries. Malaria risk stratification has been key to the targeted rollout of malaria intervention in these countries ^8^.

Malaria burden stratification is a process that involves classification of the geographical areas or localities according to factors that determine receptivity and vulnerability to malaria transmission ^10^. Some have argued that the malaria risk stratification is the first step to planning prevention and control ^10^. Malaria burden stratification is important because it allows public health officials to target their interventions to areas where they are most needed ^11^. By identifying areas with the highest burden of malaria, officials can prioritize resources and interventions to those areas, which can help to reduce the overall burden of the disease. Additionally, by monitoring changes in malaria burden over time, officials can assess the effectiveness of their interventions and make adjustments as necessary. Furthermore, Malaria burden stratification can also help to identify areas where other health issues may be present, such as poverty, poor access to healthcare, or poor housing conditions, which may need to be addressed in order to effectively combat malaria ^8^. There are no standard methods for malaria burden stratification. Examples from countries that have done the process involve using one or more burden indicators, such as prevalence, incidence, mortality, and intervention coverage indicators ^12– 14^. WHO encourages countries to define their own contexts and indicators and track their own progress ^15^.

In October 2022, the Malawi NMCP for the first time set up a task force to perform a malaria burden stratification to inform the new strategic plan to be developed as well as the Global Fund grant application scheduled in January 2023.

The following were the objectives/deliverables/terms of reference for the task force.

- Review malaria burden and intervention coverage data to describe the status to date.
- Define and document the process for malaria burden stratification and all the considerations.
- Decide on the appropriate administrative level for stratification and action, with all the considerations, including health systems factors, current administrative structures, data quality, etc.
- Stratify Malawi into appropriate burden strata and define/describe the context-specific issues driving transmission in hot spot areas.
- Recommend both current and additional interventions in each of the strata.

## Methods

### Ethical statement

In the workshop, we utilised secondary aggregate survey data and did not require ethical approval since the routine health information systems data belongs to the Ministry of Health and was analysed at an aggregate level. For prevalence data, the authors confirmed that the relevant institutional review boards in Malawi had approved the original sampling. Furthermore, the Ministry of Health granted approval to extract the data. Participants in the workshop provided their consent by accepting an invitation sent to them via email by the Ministry of Health. The email provided information about the workshop, including its objectives and expected outcomes.

### Study design

The malaria burden stratification task force set up by the NMCP comprised 18 members from the control programme comprising of members of the NMCP, NMCP partners, academia, research, district health representatives, and frontline health care workers. These members were selected in an open process during a malaria monitoring and evaluation Technical Working Group (TWG) meeting that took place on the 21 ^st^ – 23 ^rd^ of October 2022. This was a regular NMCP TWG meeting. The criteria for selecting taskforce members included willingness to participate in the meetings that the taskforce would set, extensive knowledge of the malaria landscape, membership in the NMCP for some members, and a background in academia or research related to malaria. Representation of district health services and frontline health care workers. After suggestion of names fitting into the above criteria, the final composition of the taskforce was voted by the members of the TWG. This meeting was followed by a preliminary burden stratification task force meeting held on 4 ^th^ November 2022 to select the task force leadership and plan the workshop. Similar inclusion criteria for selection of leadership was used, followed by a final vote was used to select the leadership was used.

A four-day malaria burden stratification workshop took place from 29 ^th^ November to 2 ^nd^ December 2022 in Blantyre, Malawi. In addition to the core task force members, other key malaria stakeholders who were partners with the NMCP and were known to be interested in the stratification work were invited to attend the meeting. Of note was a member from the Tanzania NMCP who was asked to share experiences from their recently conducted malaria stratification exercise. Additionally, a team from the Imperial College London shared some background information on modelling where they explained how models help in knowing where interventions can have an impact, in evaluating interventions, developing strategies and helping in- country planning. A member the PATH/MACEPA ^15^ in Seattle was with the team throughout the workshop to support the data analysis process.

### Data assembly and their sources

All essential data for malaria burden stratification was collated before the workshop for initial checks of data quality parameters. Table 1 below shows the data elements gathered before the workshop and their sources. These sources were selected as the repositories where the NCMP hosts their data on each subject area, for example malaria case data, and were therefore the inherent choice of data sources for this study. Data was collected by downloading the relevant data element from each NCMP source.

**Table 1.** Data sources.

Data element	Source
Malaria-confirmed case data aggregated at national, district and health facility levels for 2016 to 2022.	Health Information Management System (HMIS), DHIS2) ( https:// dhis2.health.gov.mw/dhis-web-commons/security/login.action)
All-cause mortality data aggregated at national, district and health facility levels for 2016 to 2022 stratified as under five and over five.	DHIS2
Population data, projected from the national 2018 census	National Statistics Office (NSO)
Prevalence data: National and regional prevalence Subnational prevalence estimates (PfPR2-10) from models developed in-house, detailed methodology available elsewhere. Estimates available for the years 2000 to 2021	Malaria Indicator Survey (MIS) Reports (2010,2012,2014,2017,2021) Chipeta *et al.* ^16^, Mategula *et al.* (manuscript in draft)
Malaria intervention coverage data on LLINS, IRS, MIP	DHIS2
Malawi Admin,0,1 2 shapefiles	NSO ( http://www.nsomalawi.mw/)
Health facility catchment shapefile, developed inhouse, methodology available in detail elsewhere	Kalonde, Mategula, Chirombo, *et al.* (manuscript in draft)

### Data quality

***HMIS data***. For most of the data quality indicators, the NMCP is on track with its routinely collected data in the demographic health information systems (DHIS2). Data quality checks performed before the meeting showed how substantial improvement in quality parameters as of 2022 compared to the baseline parameters in 2016. Table 2 below shows the proportions of data quality indicators for the routine HMIS data used in the analysis.

**Table 2.** HMIS data quality indicators 2016–2022.

		2016	2017	2018	2020	2021	2022	Target
Data Quality	indicator							
Completeness	% monthly malaria reports submitted of all expected reports	91.7%	92.4%	97.0%	92.51%	96.92%	98.32%	95%
Timeliness*	% Monthly malaria reports submitted by the 15th of next month of all expected reports.	53.9%	55.2%	77.2%	40.51%	87.34	91.60%	95%
Accuracy	% of submitted monthly malaria reports that can be validated 100%	7%	6%	40%	92	95.5	97%	60%

**Figure 1.**
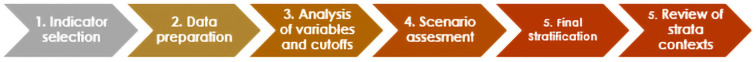
The process followed in malaria burden stratification.

Several steps were taken to come up with the strata as shown in Figure 1 above. All processes were transparent, and there was consensus before moving to each step. First, indicators were selected, and then the data was prepared into a database for analysis. These indicators were agreed prior to the meeting. Workshop participants reviewed malaria burden and intervention coverage data to describe the current status. With this process, relevant cut-off thresholds for each indicator were agreed upon, some of which were data-driven using a quartile approach, and others, such as prevalence cut-offs, were based on literature ^15^. After thoroughly reviewing the pros and cons of each prospective cut-off threshold, members reached a consensus. They decided on the appropriate administrative level for stratification and action as a district, with all the considerations including health systems factors, current administrative structures, data quality etc., and that the review of this process would be repeated on an annual basis.

To classify the districts into burden strata, the team reviewed two scenarios as shown in Table 3 below. Both scenarios used a composite score approach derived from three key variables: Scenario one prioritized districts based on incidence, prevalence, and all-cause mortality rate; whereas scenario two prioritized districts based on total cases, prevalence, and total all-cause mortality. Scenario two has been used by WHO before when determining the high burden high impact countries ^17^.

**Table 3.** Burden cut-offs and scenarios.

Strata	Scenario one (adjusts for population denominators)			Scenario two (considers caseload and mortality case load without adjusting for population)		
	Incidence	Prevalence	All-Cause Mortality Rate (Under 5)	Total Cases	Prevalence	All-Cause Mortality Totals (Under 5)
Highest Burden cutoffs	>500 cases/1000	>0.2 PfPR 2-10	>170 deaths per 100K	>300,000 cases	>0.2 PfPR 2-10	> 150 total deaths
High Burden cutoffs	200-499 cases/1000	0.1-0.2 PfPR 2-10	115-170 deaths per 100K	200,000- 300,000 cases	0.1-0.2 PfPR 2-10	100-150 total deaths
Moderate burden cutoffs	<200 cases/1000	<0.1 PfPR 2-10	<115 deaths per 100K	< 200,000 cases	<0.1 PfPR 2-10	<100 total deaths

Workshop members determined the final stratification list. It was agreed to have three cut-offs and four strata levels: highest, high, moderate, and low. The team decided to use district level and to understand contextual factors per facility for districts with high burden. In order to achieve this the members shared tasks amongst themselves to consult district teams to obtain contextual information.

The four strata are defined in Table 4 below.

**Table 4.** Malaria strata definitions.

Strata level	Definition
Highest burden strata	At least two of the highest-burden cutoffs
High burden strata	Only one of the highest burden cut-offs
Moderate burden strata	At least two of the high-burden cut-offs and zero highest-burden strata
Low burden strata	< 2 of the high-burden cut-offs, at least one of the moderate burden cut off and zero of the highest-burden

The criteria above led to the formation of district-level strata. The district burden strata for both scenarios, one and two, were discussed by the team. The workshop members then determined the final stratification list. To combine the two scenarios, the members listed combined the lists in the two scenarios and removed any duplications. The team then collated the initial district-specific contents and contextual factors with the sub-district level data guidance.

This process was initially completed during the in-person stratification workshop using 2021 malaria data, as this was what was readily available at the necessary resolution from DHIS2. In the weeks following the workshop, the process was repeated for 2018–2020 in order to provide a more comprehensive view of past malarial trends in each district of Malawi. The analysis of historical data followed the same stratification process as was established during the in-person consultative workshop, including using the same definitions for burden strata.

## Results

**Figure 2.**
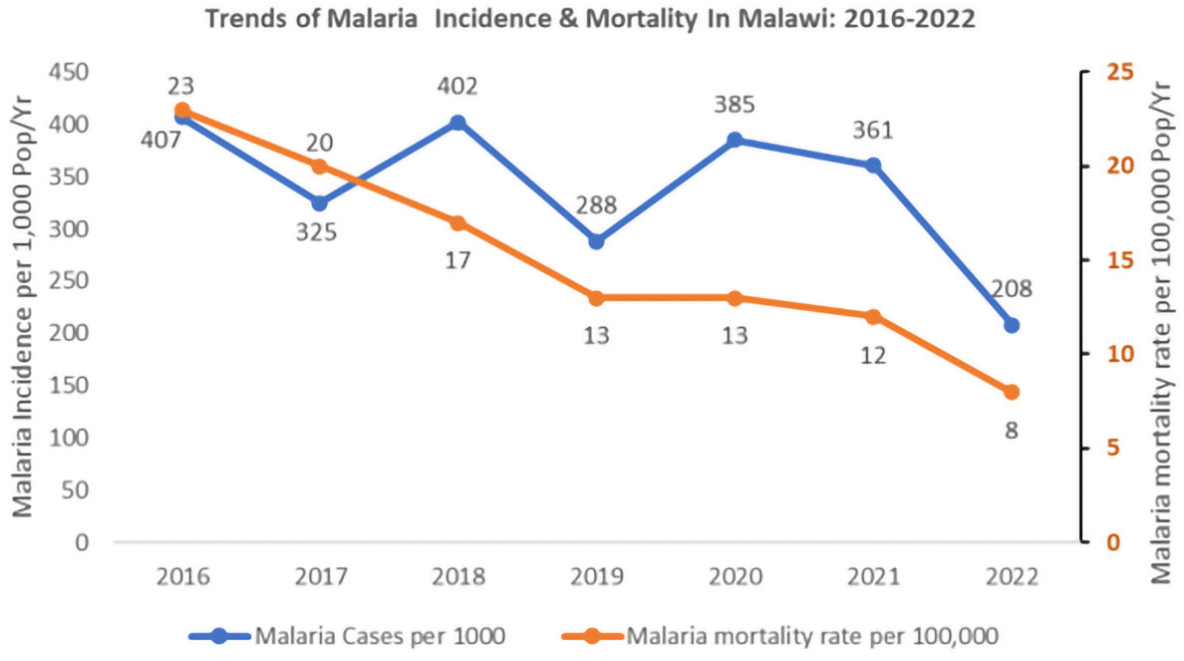
Trend of malaria incidence and mortality in Malawi 2016–2022.

A review of the incidence and mortality data showed that incidence and mortality have reduced from their baseline levels of 2016. Incidence has declined from 407 cases per 1000 in 2016 to 208 per 1000 population in 2022. Mortality has decreased from 23 per 100,000 population to 8 per 100,000 population. Figure 2 below shows the malaria incidence and mortality trends between 2016 and 2022.

**Figure 3.**
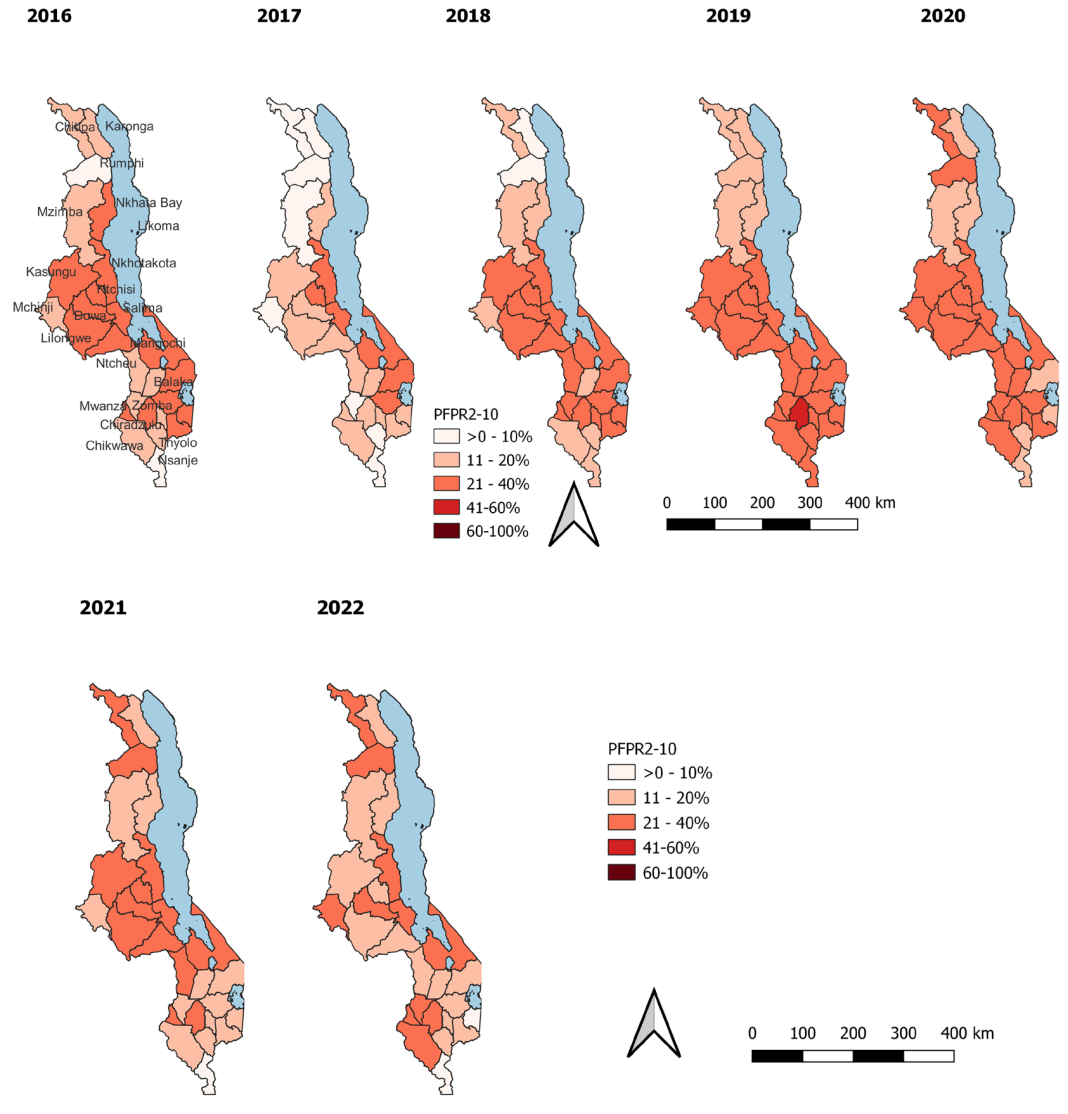
Malawi district level malaria prevalence (PfPR2_10).

Prevalence estimates for national and regional levels traditionally come from malaria indicator surveys. Subnational prevalence estimates for the age group two to 10 (PfPR2_10) have become available in the country, initially modelled by Chipeta *et al.,* (2017) and further updated by Mategula *et al.,* (manuscript in preparation) for this stratification exercise. Predicted prevalence estimates show that there is varied prevalence across Malawi, as shown in the maps below in Figure 3.

### Review of scenario one and two

**Figure 4.**
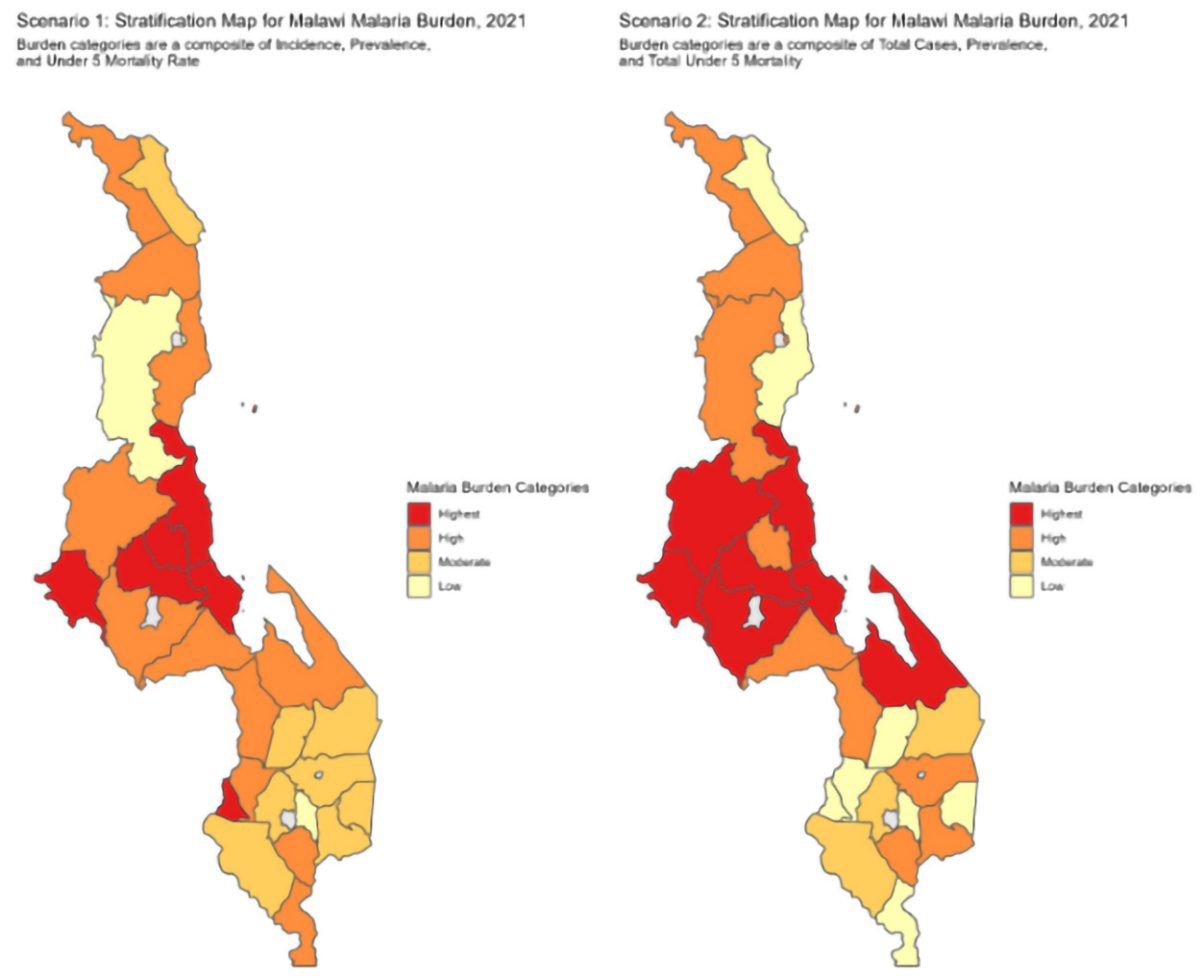
Scenario one and two for burden stratification.

Maps for the districts in the four strata based on both scenarios one and two were plotted and shown below in Figure 4.

**Figure 5.**
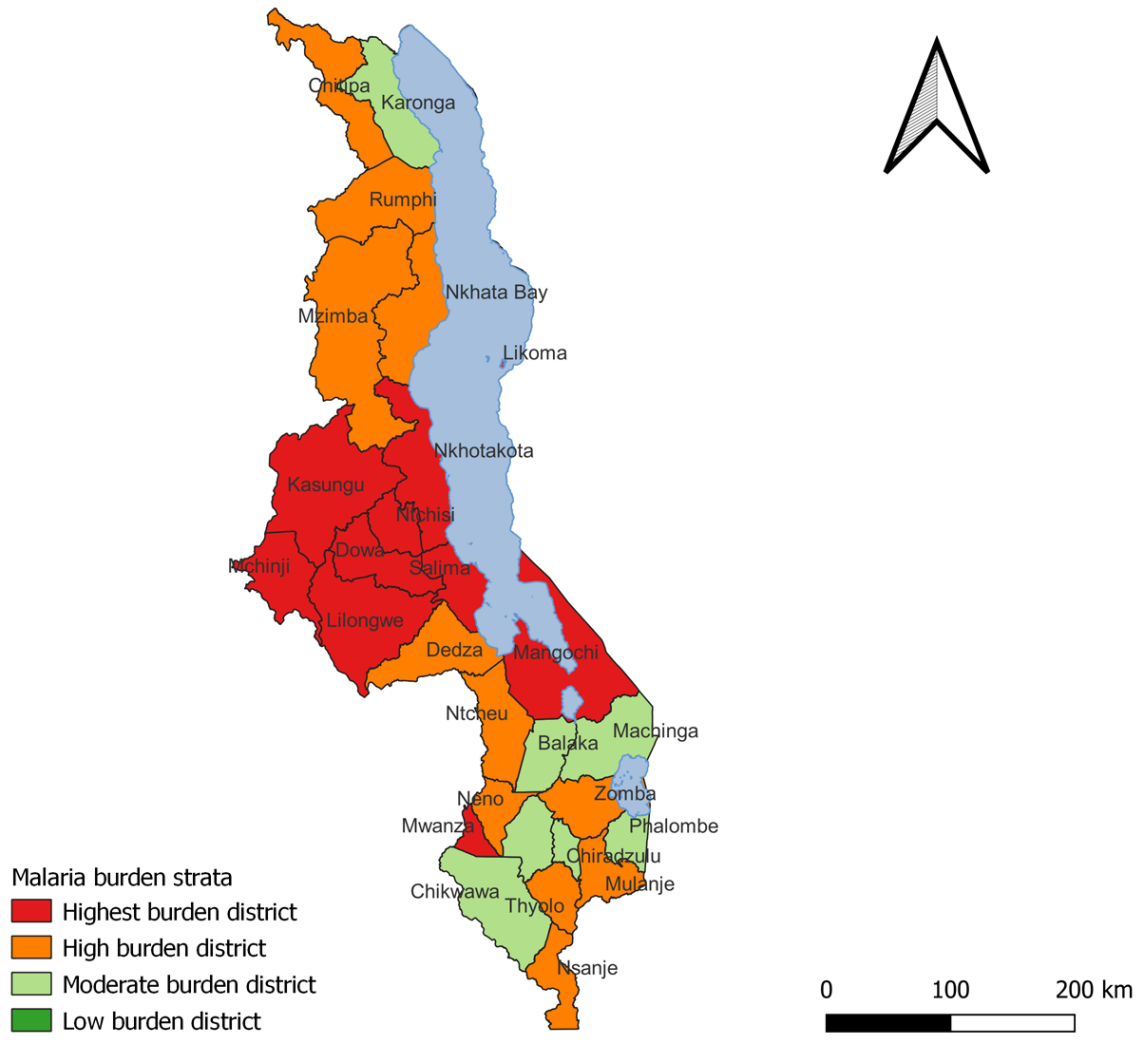
Final output for burden stratification.

The task force members agreed and endorsed the final list after reviewing scenarios one and two and combined them using the process discussed in the methodology sections ensuring that there are no duplications and that districts do not appear in two strata simultaneously. The final strata are then presented in the Table 5 below and shown in the map in Figure 5 below.

The stratification outcome concluded with 10 districts in the highest-burden stratum, 11 in the high-burden stratum, and seven in the moderate burden strata. There no districts in the low burden strata. A majority of the highest-burden districts are in the central region of the country.

**Figure 6.**
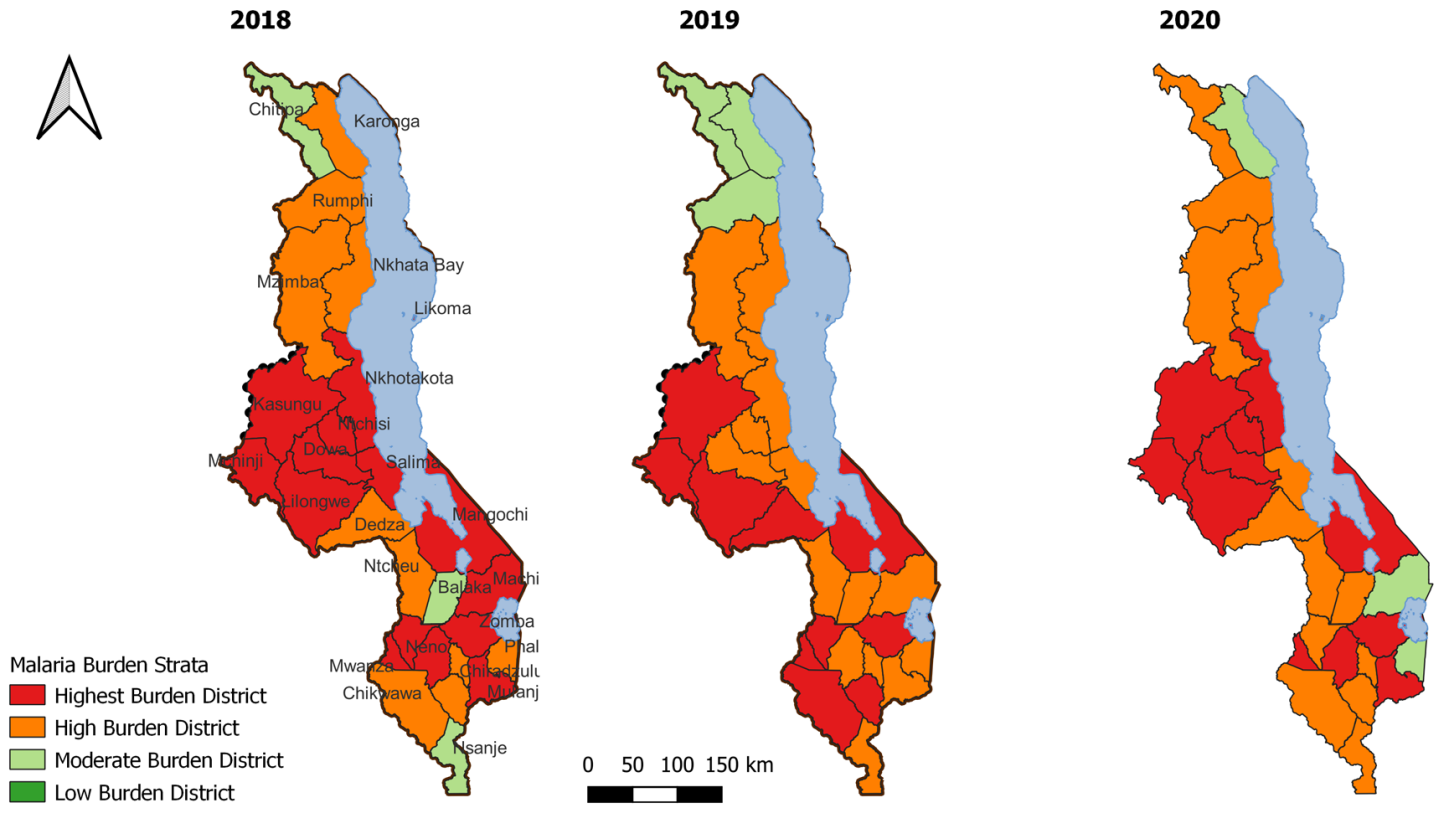
Malaria burden stratification 2018–2020.

The analysis of historical data prior to 2021 yielded similar patterns to those seen in the original 2021 stratification, albeit with higher combined burden strata in the south-western part of Malawi as shown in Figure 6 below,

**Table 5.** Lists of districts in the final stratification.

Stratum	Districts
Highest burden strata	Nkhotakota, Salima, Mchinji, Dowa, Ntchisi, Mwanza, Likoma, Lilongwe, Kasungu Mangochi
High burden strata	Chitipa, Rumphi, NkhataBay, Dedza, Ntcheu, Neno, Thyolo, Nsanje, Zomba, Mzimba Mulanje
Moderate burden strata	Karonga, Chikwawa, Balaka, Machinga, Phalombe, Blantyre, Chiradzulu
Low burden strata	No districts

## Discussion

Malawi conducted a malaria burden stratification exercise from 29th November to 2nd December 2022, using a composite of burden indicators compiled from 2021 DHIS2 data. The final stratification placed 10 out of the 28 districts in the highest-burden strata, 11 in the high-burden strata, and seven in the moderate-burden strata. There were no districts that ended up in the low-burden strata. Most of the highest-burden districts were in the country's central region, mostly those on the eastern side, along the lake, or those sharing a border with Zambia. The final stratification was expected based on the experiences of the NMCP and the frontline teams.

The next steps following this process are fully understanding the context-specific issues within the districts that drive transmission. The strata and contextual issues will then inform the intervention allocation in each stratum. This process will inform the 2023 to 2030 national malaria strategic plan. Since the NMCP is developing a Global Fund grant application for malaria control activities during the same period of the NSP development, this stratification will also justify resource allocation to the different geographical strata.

The approach used in this stratification has several strengths. Using multiple indicators to decide on the strata borrows the strengths from several indicators to develop the appropriate strata. The process of stratifying was data-driven, transparent and reproducible. This is another key strength as it makes it easy for updates to be made to the strata. We used the most recent data to inform the decisions on the strata, which has better quality indicators than historical data. WHO is encouraging country-led processes informed by the local context in dealing with health problems in low-income countries such as Malawi. This process has and continues to demonstrate in-country leadership in decision-making, effective collaboration and partnership, including the role of academics in this process.

The task force members acknowledged several limitations with the process, including the challenges with denominator data based on predictions, and sometimes has a larger variance than that of headcount for the facilities. While the most recent data was of better quality than data from previous years, we acknowledge that making decisions on one year of data can have challenges even though it is recent. As an addendum to the original workshop stratification process, we analysed historical malaria burden data from 2018–2021 to present past malarial trends at a subnational level. These historical district strata will be analysed in combination with past intervention use to inform future updates of the national stratification process, to ensure that a true malaria burden baseline is considered when considering future intervention targeting.

The task force has identified several next steps, including updating facility-level strata based on the same process and incorporating past vector control interventions to determine a baseline. Additionally, the task force will include community health care data commonly known as health surveillance assistants (HSAs) capability to handle malaria community case management. The task force will also prioritise and allocate future interventions and note criteria for their use (i.e., what level of historical burden/intervention effort/insecticide resistance would you want to target each type of intervention going forward). Furthermore, there will be a pulling out of the population in each scenario high/medium/low burden districts to determine the costs of targeting interventions. There will also be a determination of what additional analyses and modelling exercise the NMCP would be interested in to allow partners to support their implementation.

## Conclusion

For the first time, the Malawi National Malaria Control Programme has developed a malaria burden stratification to open up the use of a more targeted malaria strategy. Further next steps will allocate interventions in each stratum. We recommend maintaining the agreed method in further stratification exercises, including microstratification at the health facility level that could be done subsequently.

## Data Availability

The malaria case, mortality, and population data used in the analysis can be made available upon request, as it is under licence by a third party, the Malawi Ministry of Health, and requires permission to access from the DHIS2. Procedures to access the data can be found here. ( https://dhis2.health.gov.mw/dhis-web-commons/security/login.action). Where necessary, the corresponding author can be contacted to facilitate these requests. Prevalence data is hosted by the National Malaria Control Programme and can obtained upon request. Email requests can be made to the NMCP director ( lumbani2001@yahoo.com). Malaria indicator survey datasets for the years 2010, 2012, 2014, and 2017 are available from the DHS website. The reprocess to request the datasets is outlined here ( https://dhsprogram.com/data/Using-Datasets-for-Analysis.cfm).
