# Supplementary material for: Malaria Burden Stratification in Malawi- A report of a consultative workshop to inform the 2023-2030 Malawi Malaria Strategic Plan
Source: Wellcome Open Res. 2023 Apr 19;8:178. [Version 1] doi: 10.12688/wellcomeopenres.19110.1 (PMC10432890; doi:10.12688/wellcomeopenres.19110.1)
Supplement: Peer review history

# Peer review: Reviewer response for version 1

**DOI:** 10.21956/wellcomeopenres.21186.r61200

**Published:** 2023-08-16

**Authors:** Cara Smith Gueye

**Affiliations:** 1 University of California, San Francisco, San Francisco, California, USA

This paper describes the process of malaria burden stratification undertaken by the NMCP of Malawi. Documentation of this process is of interest to the malaria community, as this stratification process is rarely described in the literature and is useful for other NMCPs, donors and partners. I have detailed below some instances where copy editing is required to make sure readers understand the message, and a few areas where more information or detail would improve understanding of the process. The integration of epidemiological and entomological data in processes such as this burden stratification rarely occurs, so I have pointed out a few places (e.g., contextual factors) where there is an opportunity to address this, if indeed ento factors will be part of this process.

Introduction:

1. This section may need a rapid copy edit to make sure there are no errors or repetition, and that punctuation is correct.
2. Paragraph 1: Incidence and mortality goals are for 2022 – since it is now halfway into 2023, are there updates on these goals, e.g., were they reached in 2022?
3. Paragraph 3: “…application of malaria interventions is without geographic targeting…” – please remove “is”.
4. Paragraph 3: It would be interesting to have a little more information about the reduced donor/domestic funding for malaria control/elimination in Malawi. Also, it would improve the flow to find a different way to word “flattening and reducing donor support” as it is repeated a few times.
5. Paragraph 3: It would be very interesting to hear more about how the program learned through the RTS,S vaccine rollout the need to shift to a targeted intervention approach. Can the authors provide perhaps 1-2 examples?
6. Paragraph 4: In the first two sentences, malaria burden stratification and malaria risk stratification are both mentioned. It would be helpful for the reader if malaria risk stratification is explained if it is considered to be a different process than malaria burden stratification.
7. Paragraph 4: “no standard methods for malaria burden stratification” – we find this to be true as well, and a challenge for NMCPs, which is why this paper is useful to the field.
8. Paragraph 5: The authors might consider moving the task force description and TORs to the methods section.
9. At the end of the introduction: I would like to see the authors explain the rationale for writing up this process (e.g. Task force development, burden stratification) and sharing it with the malaria community. I assume that the rationale is at least partly because there are seemingly no standard methods for this stratification process and yet NMCPs need to better allocate fewer resources to achieve larger-scale goals, such as elimination.

Methods:

1. Study design:
  1. It would be interesting to know a little bit more about the TWG – what are its main goals? When was that formed? Other NMCPs may like to know about the TWG.
  2. “Representation of district health services…” – this is an unfinished sentence, please review. Also, the last sentence of this paragraph repeats “was used”.
2. Table 1:
  1. Was it possible to collate malaria confirmed case data disaggregated by age, gender, and possibly other indicators, such as occupation? It would be interesting to see any trends in cases 14 and up, for example, especially in males. Especially in lower burden areas approaching elimination, programs tend to see more adult males with a disproportionate share of the burden, and they are often harder to access.
  2. I’m also curious if and when entomological indicators were factored in, such as primary vectors, biting habits (e.g., indoor vs outdoor). These data may be brought in later as contextual data. We are just eager to see programs integrate their epidemiological and entomological data for intervention planning.
  3. I think more detail on the prevalence modeling data would be helpful to the reader. Also if not described elsewhere, what the schedule of prevalence surveys is currently in Malawi, whether national or subnational.
3. Figure 1: this figure is referred to as the “strata” in the paragraph below, when it seems to be a figure indicating the process.
4. Table 3: In the narrative it says that the team “reviewed two scenarios,” however it is unclear how these scenarios were originally developed. Were they found in the literature or guidance documents? I see that Scenario 2 was used by WHO to determine HBHI countries, but it’s unclear where Scenario 1 originated.
5. I’m also interested to read more about how these different data sources were weighed to come up with a stratification system – did the task force weigh certain data heavier than others? How did the task force integrate or triangulate across data sources? It would be interesting to know more about this process especially in relation to modeled prevalence.
6. Contextual information: I think here in the Methods section it would be good to have a full description of the type of contextual information that was sought from the district teams/health facilities. Per my above comment, I’m very interested to know if entomological data/indicators were brought into the analysis. I believe the only time contextual factors are mentioned again is in the Discussion, which means that contextual factors seemingly were not a part of this analysis, is that correct? Perhaps the role of contextual factors needs to be clearer in the methods section.

Discussion:

1. It would be interesting to hear if the NMCP was surprised by any of the strata or particular districts that were assigned a strata, based on their experience and expertise.
2. The readers may like to hear more about the types of interventions that will be targeted to the different strata, but that may be outside the scope of this paper.

Is the work clearly and accurately presented and does it cite the current literature?

Yes

If applicable, is the statistical analysis and its interpretation appropriate?

I cannot comment. A qualified statistician is required.

Are all the source data underlying the results available to ensure full reproducibility?

Yes

Is the study design appropriate and is the work technically sound?

Yes

Are the conclusions drawn adequately supported by the results?

Yes

Are sufficient details of methods and analysis provided to allow replication by others?

Partly

Reviewer Expertise:

malaria; malaria programming; malaria elimination; qualitative research methods;

I confirm that I have read this submission and believe that I have an appropriate level of expertise to confirm that it is of an acceptable scientific standard.

# Peer review: Reviewer response for version 1

**DOI:** 10.21956/wellcomeopenres.21186.r61204

**Published:** 2023-08-16

**Authors:** Mukumbuta Nawa

**Affiliations:** 1 Levy Mwanawasa Medical University, Lusaka, Zambia

**Overview:**

 The article reports on an innovative approach by the Malawi Malaria Control Program (NMCP) to stratify districts by malaria disease burden based on a combination of key indicators such as malaria incidence, prevalence and all-cause under-five mortality in children aged two to 10 years old. The exercise aims to target appropriate interventions based on the malaria burden as guided by the World Health Organisation Global Technical Strategy for Malaria Elimination 2017 – 2030. The paper describes the objectives and methodologies that were undertaken during this exercise. It adequately identified all stakeholders involved, and the Tanzanian National Malaria Control Program did another similar stratification exercise. Other stakeholders included PATH/MACEPA from Seattle, USA, the Imperial College London, UK and Liverpool School of Tropical Medicine, Liverpool, UK.

 Using data from Malawi Malaria Indicator Surveys, District Health Information System (DHIS-2) and malaria modelling data by Chipeta *et al.* 2017, they managed to stratify the 28 districts in Malawi into four strata that can be used to target malaria interventions. Of the 28 districts, ten were placed in the highest-burden strata, 11 in the high, seven in the moderate and none in the low-burden strata. The emerging pattern was that the central parts of Malawi, areas near Lake Malawi and the areas bordering Zambia had the highest malaria burden, which was also reflected and corroborated by historical data.

 Whilst the effort is innovative and commendable, there are some concerns that the authors need to address to strengthen the article further. In this regard, my overall verdict is 'approved with moderate corrections' because they may need to re-analyse some data and amend some tables and figures on top of some minor additions and edits. The main areas of concern are in the methods section, where the authors spent more time describing the stakeholders involved and the process of the workshop. They did not elaborate more on the data that was used; for example, under the sub-heading study design, they talked more about the participants of the workshop instead of surveys and DHIS2 data used, which were, to me, the true design: were these surveys adequately powered to detect prevalence and incidence respectively at their unit of analysis (district), how were the participants in the surveys enrolled. From how they gave space to participants, you would expect their results to also talk about the participants, like how many were women, average age etc. Still, they rightly talked about the data from MIS and DHIS2. Hence, the methods also need to highlight the methodologies of MIS and DHIS2 and their adequacy to represent the population and unit of analysis.

 My second moderate concern is the use of point estimates without confidence intervals; there was a wholesale qualitative interpretation of quantitative data that, for example, an increase in completeness from 92% to 98% was a substantial increase. Statistical tests must be used to test whether these were statistically significant and show the evidence using P-Values. Please see some of my specific comments:

**Specific comments by Reviewer:**

**Introduction:**

1. Paragraph one on page 4, line number 3: “The most recent strategic plan 2017 – 2022 aims to reduce …..” 2022 has passed, and this is a report in 2023 on the workshop they did in 2022; I suggest they use “aimed to reduce…”.
2. Paragraph four on page 4, lines 5-7: “In a heterogeneous transmission setting, application of malaria interventions is without geographic targeting at the subnational level is less cost-effective” The sentence has too many connectors and is therefore unclear. It also makes the next sentence unclear. This is where they are trying to make a case for justifying finer-level stratification to target interventions at the district level.

**Methods:**

1. Ethical statement paragraph 1 on page 5, lines 8 -10: “Participants in the workshop provided consent by accepting …” This statement is unnecessary because the workshop participants were used as fellow researchers, not respondents.
2. Study design: The narration on the study design is all about participants; I feel this narration can be placed under a sub-title workshop process or participants. The study design should highlight the MIS surveys and DHIS2 routine data.
3. Data quality page 5, lines 2-3: “The NMCP is on track with its routinely collected data in the Demographic Health Information System (DHIS2)”. The DHIS2 is an open-source software developed by the University of Oslo. It is deployed in many countries and is called District Health Information System. Kindly change this.
4. Data quality page 5, lines 3-6. “Data quality checks showed substantial improvement from 2016 to 2022”. This is my main area of concern regarding the analysis of this article; the authors analyse point estimates and make qualitative inferences without statistical testing of the data; for example, a completeness of 92% in 2016 may not be statistically significant compared to a completeness of 98% in 2022. The accuracy of 7% is very likely to be statistically significant compared to 97% in 2022. I suggest they insert a column with a P value to justify this interpretation.

**Results:**

1. Table 2 on page 6:
  The table needs to be cleaned up; some percentages have the sign (%) while others do not.
  Secondly, some numbers have two decimal places, while others have none. Given the wide range from 7% to 98%, I propose that the authors consider not using decimal places.
2. Figure 2 on page 7. For the use of point estimates on the line graph on malaria trends, I suggest the inclusion of confidence intervals in the graph. This will be more informative and give confidence to the interpretation.

**Discussion:**

1. The whole discussion seems to be a one-way narrative from the author’s perspective. I suggest the authors compare and contrast with other existing literature to give a more scholarly context. For example, PMI and GFATM have funded a lot of interventions such as IRS, LLIN mass distribution and community health workers in sub-Saharan Africa; some of these activities have been published and can be cited as they are discussing their findings.
2. Because of not comparing and contrasting highlighted in point number 9 in the discussion, the list of references is only 17; it may be good to increase the number of references to between 25 – 30.

Is the work clearly and accurately presented and does it cite the current literature?

Partly

If applicable, is the statistical analysis and its interpretation appropriate?

Partly

Are all the source data underlying the results available to ensure full reproducibility?

Yes

Is the study design appropriate and is the work technically sound?

Partly

Are the conclusions drawn adequately supported by the results?

Partly

Are sufficient details of methods and analysis provided to allow replication by others?

Yes

Reviewer Expertise:

Epidemiology and Biostatistics in the Area of Malaria

I confirm that I have read this submission and believe that I have an appropriate level of expertise to confirm that it is of an acceptable scientific standard, however I have significant reservations, as outlined above.

# Peer review: Reviewer response for version 1

**DOI:** 10.21956/wellcomeopenres.21186.r61206

**Published:** 2023-07-17

**Authors:** Fekadu Massebo

**Affiliations:** 1 Arba Minch University, Arba Minch, Southern Nations, Nationalities, and People's Region, Ethiopia

The authors provided robust malaria stratification information and grouped the districts in the country into three categories with the highest, high, and moderate burden; however, no districts with low malaria burden were discovered. The hypothesis of malaria burden stratification and recommendation for targeted intervention in a country where 75% of districts have either the highest or high burden of malaria remains unverified.

**Introduction:**

 General comments:

- The authors attempted to establish the need of malaria stratification; however they did not argue how malaria payback is harmful when intervention coverage and intensity are lowered. The last two years (21/22) have shown that reducing malaria to a significant level takes decades, whereas the payback is short when there are gaps in action.
- In a country where 75% of districts fall under a high burden, how are heterogeneous transmissions defined? 
- Although the authors investigate district-level stratification and malaria transmission heterogeneity, they don't recognise village/household-level transmission heterogeneity, as well as the limitations and advantages of each.
- Why do the authors focus on the aggregate data at district level? Does it make more sense to incorporate data from each village rather than district-level aggregate data to identify contribution each village that make bias due to the district level aggregate data?
- Why didn't the authors discuss the issues of data quality and how they dealt with it?
- Why do the authors conclude that the NMCP should begin targeted action utilising stratification based on aggregate data and recommending further stratification using health facility data?

Is the work clearly and accurately presented and does it cite the current literature?

Yes

If applicable, is the statistical analysis and its interpretation appropriate?

Partly

Are all the source data underlying the results available to ensure full reproducibility?

Yes

Is the study design appropriate and is the work technically sound?

Partly

Are the conclusions drawn adequately supported by the results?

Partly

Are sufficient details of methods and analysis provided to allow replication by others?

No

Reviewer Expertise:

Malaria and vector control interventions focusing on housing and animals

I confirm that I have read this submission and believe that I have an appropriate level of expertise to confirm that it is of an acceptable scientific standard, however I have significant reservations, as outlined above.
